# Association of Clinical Signs and Symptoms with Abnormal Urinalysis Findings of Blunt Trauma Patients; a Cross-Sectional Study

**Published:** 2019-11-11

**Authors:** Bahram Zarmehri, Ayeh Shouman, Elham Pishbin, Niaz-Mohammad Jafari Chokan, Mona Najaf Najafi, Seyed Reza Habibzadeh, Esmaeil Rayat Dost, Mahdi Foroughian

**Affiliations:** 1 Department of Emergency Medicine, Faculty of Medicine, Mashhad University of Medical sciences, Mashhad, Iran.; 2 Clinical Research Unit, Mashhad University of Medical Sciences, Mashhad, Iran.; 3 Department of Emergency Medicine, Jahrom University of Medical sciences, Jahrom, Iran.

**Keywords:** Urinalysis, urogenital system, hematuria, multiple trauma, signs and symptoms

## Abstract

**Introduction:**

Urinalysis (UA) is performed routinely as a diagnostic screening test for trauma patients in most centers. This study aimed to examine the relationship between patients’ clinical signs and symptoms with UA findings.

**Methods:**

This cross-sectional study was carried out on multiple trauma patients between 18 to 65 years old, who were referred to the Emergency Department. UA was performed for all patients and its association with clinical signs and symptoms (pain, tenderness, abrasion, ecchymosis, hematoma, etc.) in abdomen, back, flank, and inferior hemi-thorax was evaluated.

**Results:**

640 patients with the mean age of 39.8 ± 11.2 years were studied (65.0% males). 271 (42.4%) cases had associated injuries and 554 (86.6%) cases had at least one sign or symptom of trauma in abdomen, back, flank or inferior hemi-thorax. 146 (22.8%) patients had negative UA. Among cases with positive UA, 364 (56.9%) cases had microscopic hematuria with RBC < 25/HPF, 60 (9.4%) had microscopic hematuria with RBC ≥ 25/HPF and 70 (10.9%) had gross hematuria. None of the asymptomatic patients had microscopic hematuria with RBC ≥ 25/HPF and gross hematuria (p <0.001). Symptomatic patients who had signs in the abdomen, back or inferior hemi-thorax mainly had microscopic hematuria with RBC < 25/HPF, but those with signs in the flank, mainly had microscopic hematuria with RBC ≥ 25/HPF (p<0.001). Patients with pain, tenderness, abrasion, and ecchymosis in flank had a higher risk of positive UA findings (figure 2; p <0.001).

**Conclusion:**

Based on the findings of the present study, patients with any findings of pain, tenderness, abrasion, or ecchymosis in flank had higher risk of abnormal UA and perhaps urogenital injuries. None of the asymptomatic patients had microscopic hematuria with RBC ≥ 25/HPF and gross hematuria.

## Introduction

Trauma is the main cause of mortality in people aged 1-44 years (1). It accounts for more than 6 million deaths, yearly (2). Trauma related injuries are the main cause of long-lasting morbidity and disability, especially in young patients with their productive years ahead (3, 4). Although in the last five decades management of trauma patients has advanced, trauma remains a serious health problem in all societies with different economic, social and health conditions (5). 

According to previous studies, abdomen is the third most common site in the body that requires surgical intervention following trauma. Nevertheless, the evaluation and diagnosis of intra-abdominal injury is still a challenge for doctors dealing with these patients (6). Most damage to abdominal organs is caused by abdominal blunt trauma (7, 8). Abdominal trauma could include genitourinary trauma, which includes a wide range of organs such as kidneys, ureters, bladder, urethra, penis, scrotum, and testicles (1). Kidneys are the most commonly affected organs in urinary trauma. They are injured in 5% of all traumas and in 10% of abdominal traumas (9). Renal and genitourinary trauma is seen in all age groups and in both sexes, although it is more common in men compared to women (10).

Early identification and appropriate management of genitourinary tract damage can reduce potential long-term complications, including renal failure, chronic hypertension, urinary incontinence and sexual dysfunction (11). 

Signs, symptoms and findings of clinical examinations in patients with genitourinary trauma are diverse and non-specific. These manifestations may include abdominal, rib, back, pelvic or testicular pain, urinary retention, penile and scrotal hematoma or ecchymosis and blood in the urethral meatus. 

In trauma patients, abdominal and pelvic computed tomography (CT) scan, with intravenous contrast, is the gold standard for detecting kidney injury. However, to prevent the complications of this diagnostic method, urinalysis (UA) is performed as a screening test to determine the cases requiring CT scan (10). The negative results of urine tests in most of the cases, suggest that routine performance of this test must be reconsidered. Therefore, this study aimed to evaluate the relationship between clinical symptoms and UA findings of multiple trauma patients. 

## Methods

***Study design and setting***

This cross-sectional study was carried out on multiple trauma patients, who were referred to the Emergency Department of Hashemi Nejad Hospital, Mashhad, Iran, between September 2017 and September 2018. UA was performed for all patients and the association of UA with clinical signs and symptoms was evaluated. In the beginning of the project, the aim of the study was explained to the patients and after completing the informed consent form, patients were included. This research was approved by the Committee on Organizational Ethics of the Faculty of Medical Sciences, Mashhad University of Medical Sciences (IR.MUMS.fm.REC.1396.64).

***Participants***

 Patients less than 18 years of age, those with previous renal disease (such as stones, cysts, tumors, chronic kidney disease, single kidneys), unstable hemodynamic, urinary tract infection, clear symptoms of duct injury (such as hematoma in perineum and blood at the meatus of the penis), and unreliable examination (e.g. loss of consciousness, poisoning), those who were pregnant or on their menstrual period, and patients with penetrating trauma were excluded. 

***Data gathering***

On admission to the emergency department a complete history was obtained and registered for each patient including demographic features such as gender and age, location of trauma (abdomen, flank, back, inferior hemi-thorax, and other locations), trauma mechanism (e.g. falling, vehicle crash, motorcycle, pedestrian crash, direct abdominal trauma, direct back trauma, direct inferior hemi-thorax trauma, sudden impact injury), signs and symptoms (including pain, tenderness, abrasion, ecchymosis and hematoma in the abdomen, back, flank and inferior hemi-thorax), and associated injuries (inferior rib fracture, thoracolumbar vertebral fracture, pelvic fracture, long bone fracture, Intra-abdominal bleeding). Information about each patient was recorded using appropriate codes.

UA was requested within 24 hours after trauma. Urine test results were divided into 4 separate categories including negative UA, gross hematuria, microscopic hematuria with RBC (Red Blood Cells) <25/HPF and microscopic hematuria with RBC ≥ 25/HPF. All of these data were collected and put in a checklist. The urine test was interpreted by a blinded laboratory technician. A trained emergency medicine resident was responsible for data gathering.

***Statistical analysis***

Data were analyzed via SPSS version 16 software. Demographic data were presented using descriptive statistical methods, including central indicators, distribution and frequency distribution, in the form of appropriate tables and charts. ANOVA test was used to compare quantitative variables between the four groups (based on the results of UA). Chi-Square test was used to compare qualitative variables between the four groups. In all calculations, p-value of 0.05 was considered as the level of significance.

## Results

***Baseline characteristics of studied patients***

A total of 640 patients with the mean age of 39.8 ± 11.2 years were studied (65.0% males). Table 1 summarizes the baseline characteristics of studied patients. The most frequent mechanisms of trauma were pedestrian-vehicle accident (39.8%), motorcycle accident (34.4%), and falling (19.2%). 271 (42.4%) cases had associated injuries and 554 (86.6%) cases had at least one sign or symptom of trauma in abdomen, back, flank or inferior hemi-thorax. Distribution of clinical findings in mentioned locations are presented in table 2.

***UA and clinical symptoms***

146 (22.8%) patients had negative UA. Among cases with positive UA, 364 (56.9%) cases had microscopic hematuria with RBC < 25/HPF, 60 (9.4%) had microscopic hematuria with RBC ≥ 25/HPF and 70 (10.9%) had gross hematuria. 

**Figure 1:**
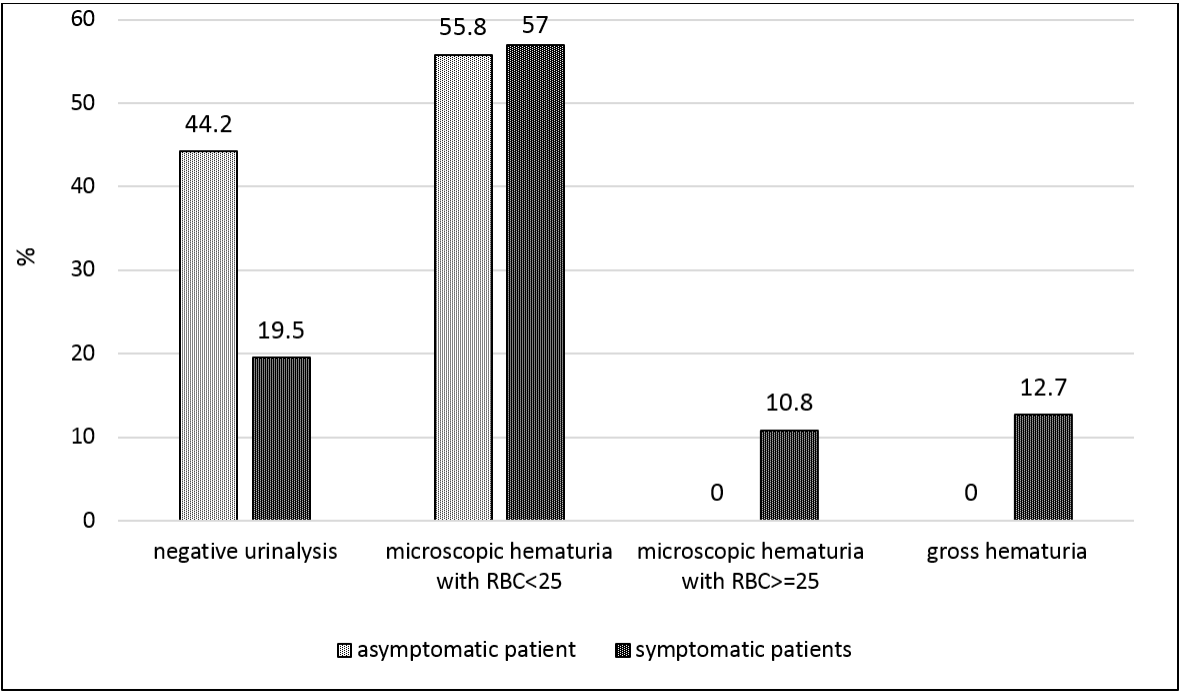
Distribution of urinalysis findings based on presence or absence of symptoms (pain, tenderness, abrasion, hematoma, etc.) in abdomen, back, flank or inferior hemi-thorax. RBC: red blood cell count per High Power Field

There was not any significant relationship between age (p = 0.83), gender (p = 0.83), mechanisms of trauma (p = 0.29), or presence of associated injuries (p = 0.456) and UA findings. Figure 1 and table 3 show the distribution of UA findings based on presence or absence of symptoms. None of the asymptomatic patients had microscopic hematuria with RBC ≥ 25/HPF and gross hematuria (p <0.001).

**Figure 2:**
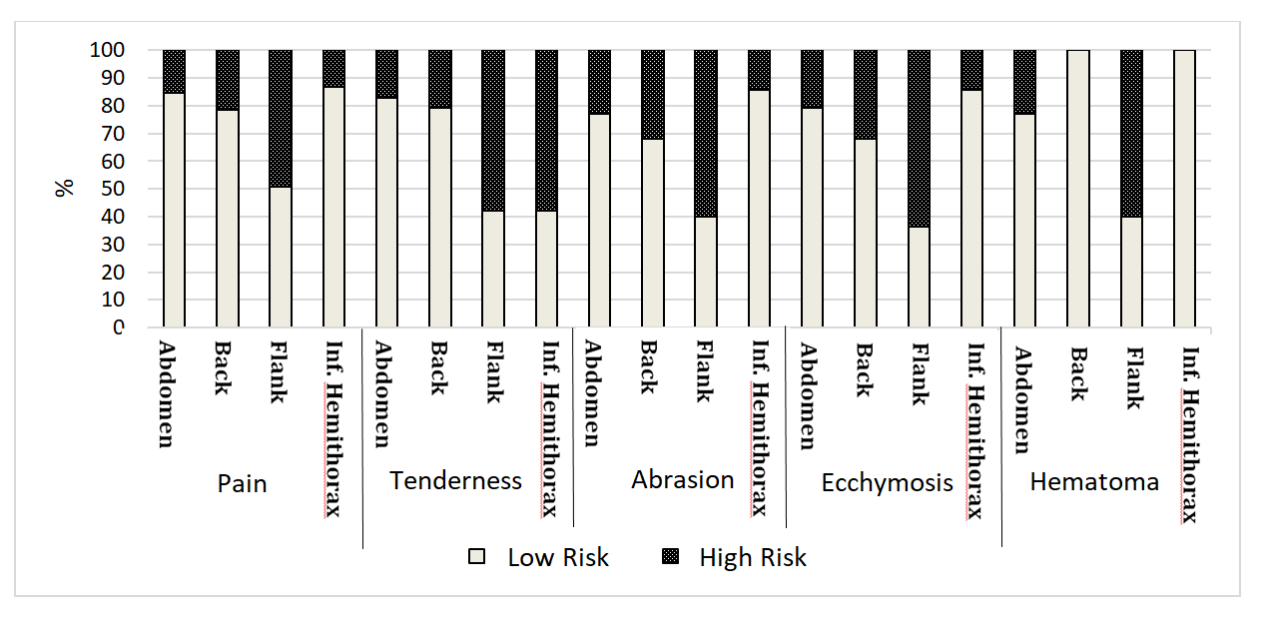
Association of clinical signs and symptoms with the risk of abnormal urinalysis findings (p < 0.001)

Symptomatic patients who had signs in the abdomen, back or inferior hemi-thorax mainly had microscopic hematuria with RBC < 25, but those with signs in the flank, mainly had microscopic hematuria with RBC ≥ 25/HPF (p<0.001). Patients with pain, tenderness, abrasion, and ecchymosis in flank had a higher risk of abnormal UA (figure 2; p <0.001).

**Table 1:** Baseline characteristics of studied patients

**Variables **	**Number (%)**
**Gender **	
Male	416 (65.0)
Female	224 (35.0)
**Trauma mechanism**	
Falling down	123 (19.2)
Motorcycle accident	220 (34.4)
Pedestrian-vehicle accident	255 (39.8)
Direct abdominal trauma	17 (2.7)
Direct trauma (flank, back,…)	25 (3.9)
**Associated injuries** ^1^	
Inferior rib fracture	9 (3.3)
Thoracolumbar vertebral fractures	41 (15.1)
Pelvic fractures	36 (13.2)
Long bone fractures	199 (73.4)
Intra-abdominal hemorrhage	19 (1.1)
**Clinical signs and symptoms** ^2^	
Pain	502 (90.6)
Tenderness	399 (72.2)
Abrasion	169 (30.5)
Ecchymosis	96 (17.1)
Hematoma	26 (4.6)

1: Some patients had more than one associated damage. 2: findings of abdomen, back, flank, and inferior hemithorax examination.

**Table 2:** Distribution of clinical signs and symptoms according to their location

**Location**	**Pain**	**Tenderness**	**Abrasion**	**Ecchymosis**	**Hematoma**
Abdomen	205 (40.8)	174 (43.6)	78 (46.2)	53 (55.2)	13 (50)
Back	199 (39.6)	150 (37.6)	59 (34.9)	25 (26)	2 (7.7)
Flank	83 (16.5)	62 (15.5)	25 (14.8)	11 (11.5)	10 (38.5)
Hemi-thorax	15 (3)	13 (3.3)	7 (4.1)	7 (7.3)	1 (3.8)

* Some patients had more than one clinical symptom. Data are presented as frequency (%).

**Table 3:** Distribution of patients in terms of clinical symptoms, site of injury and urinalysis

**location**	**Urinalysis**				**P **
	**Negative **	**RBC < 25**	**RBC ≥ 25**	**Gross hematuria**	
**Symptomatic patients**					
Abdomen	(24.6)52	(59.2)125	(4.7)10	(11.4)24	0.001
Back	(14.2)33	(64.4)150	(6.8)16	(14.6)34	
Flank	(21.3)20	(31.9)30	(36.2)34	(10.6)10	
Hemi-thorax	(18.8)3	(68.8)11	0	(12.4)2	
**Pain**					
Abdomen	(23.9)49	(60.5)124	(4.9)10	(10.7)22	0.001
Back	(14.1)28	(64.3)128	(7.5)15	(14.1)28	
Flank	(2.9)19	(27.8)3	(37.3)31	(12)10	
Hemi-thorax	(20)3	(66.7)10	0	(3.3)2	
**Tenderness**					
Abdomen	(25.3)44	(57.5)100	(5.7)10	(11.5)20	0.001
Back	(13.3)20	(66)99	(8.7)13	(12)18	
Flank	(12.9)8	(29)18	(48.4)30	(9.7)6	
Hemi-thorax	(7.7)1	(76.9)10	0	(15.4)2	
**Abrasion**					
Abdomen	(29.5)23	(47.4)37	(7.7)6	(15.4)12	NA
Back	(20.03)12	(47.5)28	(20.03)12	(11.09)7	
Flank	(20)5	(20)5	(44)11	(16)4	
Hemi-thorax	(28.6)2	(57.1)4	0	(14.3)1	
**Ecchymosis**					
abdomen	(22.6)12	(56.6)30	(11.3)6	(9.4)5	NA
Back	(20)5	(48)12	(20)6	(12)3	
Flank	(18.2)2	(18.2)2	(54.5)6	(9.1)1	
Hemi-thorax	(28.6)2	(57.1)4	0	(14.3)1	
**Hematoma**					
Abdomen	(30.8)4	(46.2)6	(15.4)2	(17.6)1	NA
Back	0	(100)2	0	0	
Flank	(20)2	(20)2	(30)3	(30)3	
Hemi-thorax	0	(100)1	0	0	

Data are presented based on frequency (%). RBC: red blood cell count per High Power Field.

## Discussion

Based on the findings of the present study, patients with any findings of pain, tenderness, abrasion, or ecchymosis in flank had a higher risk of abnormal UA and perhaps urogenital injuries. None of the asymptomatic patients had microscopic hematuria with RBC ≥ 25/HPF and gross hematuria.

In most centers, including ours, UA is used as a screening test to diagnose intra-abdominal injuries, including renal injury. However, the high number of normal results of this para-clinical method suggests that we need to create reliable clinical decision rules that could identify low-risk patients who do not require para-clinical examination. 

The most common mechanism of trauma in the present study was pedestrian accident (39.8%). 50% of patients with direct flank trauma had microscopic hematuria with RBC ≥ 25/HPF. This indicates the importance of this mechanism in predicting renal injury and the need for UA in this case, although there was no statistically significant correlation between injury mechanism and UA results. 

Results of UA showed that most symptomatic patients have microscopic hematuria with RBC <25/HPF, or have negative UA results. In addition, none of the asymptomatic patients had gross hematuria or microscopic hematuria with RBC≥25/HPF. This suggests that if the patient does not have clinical symptoms, UA may be discarded. Moreover, 36.2% of patients with clinical symptoms in the flank area had microscopic hematuria with RBC ≥ 25/HPF. 37.3% of the patients with flank pain, 48.4% of patients with flank tenderness, 44% of patients with flank abrasion, 54.5% of patients with flank ecchymosis, and 30% of patients with flank hematoma, had microscopic hematuria with RBC ≥ 25/HPF. The rate of microscopic hematuria with RBC ≥ 25/HPF was lower in other areas. This indicates the importance of these symptoms in the flank area. It should be noted that presence of ecchymosis and hematoma did not significantly correlate with UA results, which is probably due to the small number of patients with these symptoms in the study. In categorizing patients into two groups of high risk for renal injury (microscopic hematuria with RBC ≥ 25/HPF or gross hematuria) and low risk for renal injury (negative UA or microscopic hematuria with RBC <25/HPF), the frequency of flank symptoms including tenderness, abrasion, and ecchymosis was significantly higher in those with a high risk of renal injury. This also signifies the importance of symptoms in the flank area. It can be concluded that the symptoms in the flank area are very important, and if there are symptoms in this area para-clinical examination, including UA, is necessary to detect or rule out renal injury. 

Holmes et al. (2009) reviewed the rules that predict the risk of intra-abdominal injury to be low in patients with blunt abdominal trauma. The results showed that the use of a combination of Glasgow Coma Scale (GCS) less than 14, rib tenderness, abdominal tenderness, femoral fractures, hematuria with RBC more than 25/HPF, hematocrit less than 30%, and abnormal chest radiography had 8.95% sensitivity, 29.9% specificity and 6.98% negative predictive value in the determination of intra-abdominal injury. While, some of the criteria reviewed in this study differ from the criteria of our study, the results of the two studies are similar. Patients without the symptoms mentioned in the study had a lower risk of intra-abdominal injury (especially injuries requiring surgical intervention) and it seemed that patients would not benefit from CT scan. Note that, in this study, intra-abdominal injury was studied in general, while in our study only the predictors of renal injury were considered (12). 

Jones et al. also reviewed the value of UA in blunt trauma patients in 2017. UA was normal in 810 patients (45%). Among these 810 patients, 2 (0.2%) had genitourinary injury, but none of them required intervention. The researchers concluded that negative UA plays an important role in predicting or ruling out urogenital and intra-abdominal injuries. This helps in preventing exposure to unnecessary radiation. In this study, clinical manifestations had not been studied and the relationship of UA results with urogenital injury was examined, but in general, it is consistent with the results of our study (13). In 2016 Sabzghabaei et al examined 325 patients with abdominal blunt trauma. In this study, urine test results were normal in about half of patients. The results of CT scan of 193 patients (59.6%) were normal overall and 90% were normal for kidney injury, where 32 (10%) had kidney injury. The researchers stated that UA has a low diagnostic value in predicting intra-abdominal injury in trauma patients, and it could be used as a helpful diagnostic tool, along with other sources, such as clinical findings and imaging. Unlike our study, this study did not address the clinical symptoms, but its conclusion was confirmed by our findings as it recommended the use of clinical findings as a diagnostic tool (14). 

In the same context, Mustafa et al. (2017) studied the value of UA in patients with abdominal blunt trauma. Out of the 100 patients who participated in the study, 56 had microscopic hematuria, 17 of which had gross hematuria, and 44 had no hematuria. Most patients who had intra-abdominal injury had hypovolemic shock (OR: 8.4, CI95%: 2.7-26), abdominal wall hematoma (OR: 3.1, CI95%: 1.2-7.9), and/or anemia (OR: 3.6; CI95%: 1.2-10.3), at the time, UA was not successful in predicting intra-abdominal injury. The researchers concluded that the use of UA is not effective enough to predict intra-abdominal injury, and should therefore not be used as a key component in patients with blunt abdominal trauma. Although the clinical criteria of this study differ from our study, the results of both studies are similar and indicate the importance of clinical symptoms in comparison to UA (15). 

In 2015, Olthof et al. conducted a retrospective study, to examine the validity of UA in predicting traumatic urogenital injury. The incidence of intra-abdominal injury and urogenital injury were 13% and 8%, respectively. In this study, regardless of imaging findings, gross hematuria was detected in 73% of cases with urogenital injury, whereas microscopic hematuria was detected in only 4% of those patients. Olthof’s study, like our study, proves that UA is not an appropriate tool for predicting intra-abdominal injury. Therefore, the researchers suggested that UA should be removed from routine trials in patients with blunt trauma and should only be used in specific cases. Note that, in contrast to our study, which was intended only for renal injury, this study examined general intra-abdominal injuries (16).

Not only studies on UA have shown the importance of clinical symptoms to decide on further para-clinical examinations and the need of screening tools, other studies investigating intra-thoracic damage have also shown the importance of clinical symptoms in determining the need for further para-clinical studies, which confirms our results. 

In most centers, including our center, routine UA is requested for all multiple trauma patients. This study demonstrates the importance of clinical symptoms for determining the need for para-clinical interventions and suggests the use of clinical signs to predict renal injury, which in turn prevents the imposition of additional costs and emergency crowding. 

## Limitations

One of the limitations of this study was the lack of CT scans of participating patients due to its high costs and complications. In addition, this study only covers patients who were referred to the Emergency Department of Hashemi Nejad Hospital. Since genitourinary trauma is associated with intra-abdominal trauma in many cases, and since hematuria is one of the symptoms of intra-abdominal injury, it would have been better to exclude patients with intra-abdominal injury. On the other hand, the focus on kidney injury and clinical symptoms is one of the strengths of this study. In order to increase the validity of the results, we suggest that for future studies, in addition to the registration of symptoms and clinical signs and hematuria, the study must be multi-centered and include CT scans of patients.

## Conclusion:

Based on the findings of the present study, patients with any findings of pain, tenderness, abrasion, or ecchymosis in flank had a higher risk of abnormal UA and perhaps urogenital injuries. None of the asymptomatic patients had microscopic hematuria with RBC ≥ 25/HPF and gross hematuria.

## Authors’ ORCIDs:

**Bahram Zarmehri:** 0000-0002-8687-1821

**Ayeh Shouman:**

**Elham Pishbin:** 0000-0002-3082-8074

**Niaz-Mohammad Jafari Chokan:** 0000-0003-1872-574X

**Mona** **Najaf Najafi:** 0000-0003-4962-6787

**Seyed Reza Habibzadeh**: 0000-0003-4569-1776

**Esmaeil Rayat Dost:** 0000-0002-9430-7913

**Mahdi Foroughian:** 0000-0002-3944-9361

bahram zarmehri Email: zarmehrib@mums.ac.ir

Ayeh Shouman Email: choumane.aya.yas@gmail.com

Elham Pishbin Email: pishbine@mums.ac.ir

Niaz-Mohammad Jafari Chokan Email: drjafari521@gmail.com

Mona Najaf Najafi Email: najafnm@mums.ac.ir

Seyed Reza Habibzadeh Email: habibzadehr@mums.ac.ir

Esmaeil Rayat Dost Email: e.rayat.dost@gmail.com

Mahdi Foroughian Email: foroughianmh@mums.ac.ir
